# Proteasome Inhibition Increases the Efficiency of Lentiviral Vector-Mediated Transduction of Trabecular Meshwork

**DOI:** 10.1167/iovs.17-22074

**Published:** 2018-05

**Authors:** Zeynep Aktas, Hongyu Rao, Sarah R. Slauson, B'Ann T. Gabelt, Inna V. Larsen, Rachael T. C. Sheridan, Leonie Herrnberger, Ernst R. Tamm, Paul L. Kaufman, Curtis R. Brandt

**Affiliations:** 1 Department of Ophthalmology and Visual Sciences, University of Wisconsin-Madison, Madison, Wisconsin, United States; 2 Department of Ophthalmology, Gazi University Medical Faculty, Ankara, Turkey; 3 UW Carbone Cancer Center Flow Cytometry Laboratory, University of Wisconsin-Madison, Madison, Wisconsin, United States; 4 Institute of Human Anatomy, University of Regensburg, Regensburg, Germany; 5 McPherson Eye Research Institute, University of Wisconsin-Madison, Madison, Wisconsin, United States; 6 Department of Medical Microbiology and Immunology, University of Wisconsin-Madison, Madison, Wisconsin, United States

**Keywords:** trabecular meshwork, FIV vector, MG132, gene therapy, proteasomes

## Abstract

**Purpose:**

To determine if proteasome inhibition using MG132 increased the efficiency of FIV vector–mediated transduction in human trabecular meshwork (TM)-1 cells and monkey organ-cultured anterior segments (MOCAS).

**Methods:**

TM-1 cells were pretreated for 1 hour with 0.5% dimethyl sulfoxide (DMSO; vehicle control) or 5 to 50 μM MG132 and transduced with FIV.GFP (green fluorescent protein)– or FIV.mCherry-expressing vector at a multiplicity of transduction (MOT) of 20. At 24 hours, cells were fixed and stained with antibodies for GFP, and positive cells were counted, manually or by fluorescence-activated cell sorting (FACS). Cells transduced with FIV.GFP particles alone were used as controls. The effect of 20 μM MG132 treatment on high- and low-dose (2 × 10^7^ and 0.8 × 10^7^ transducing units [TU], respectively) FIV.GFP transduction with or without MG132 was also evaluated in MOCAS using fluorescence microscopy. Vector genome equivalents in cells and tissues were quantified by quantitative (q)PCR on DNA.

**Results:**

In the MG132 treatment groups, there was a significant dose-dependent increase in the percentage of transduced cells at all concentrations tested. Vector genome equivalents were also increased in TM-1 cells treated with MG132. Increased FIV.GFP expression in the TM was also observed in MOCAS treated with 20 μM MG132 and the high dose of vector. Vector genome equivalents were also significantly increased in the MOCAS tissues. Increased transduction was not seen with the low dose of virus.

**Conclusions:**

Proteasome inhibition increased the transduction efficiency of FIV particles in TM-1 cells and MOCAS and may be a useful adjunct for delivery of therapeutic genes to the TM by lentiviral vectors.

Primary open-angle glaucoma (POAG) is a leading cause of irreversible blindness worldwide.^1,2^ The major mechanism for increased intraocular pressure (IOP) in POAG is decreased aqueous humor outflow via the trabecular meshwork (TM)/Schlemm's canal (SC)/collector channels (CCs)/episcleral veins (EVs) that collectively comprise the conventional outflow pathway.^3,4^ The major resistance seems to lie near the interface of the juxtacanalicular meshwork (JCT) and the inner wall SC endothelium, with perhaps additional resistance in the distal outflow pathway comprising the SC outer wall, CCs, and EVs.^5^ Several drugs approved for ocular hypotensive glaucoma therapy exert their outflow resistance-reducing effects on the conventional outflow pathway, specifically at the JCT/SC inner wall. Rho kinase inhibitors,^6–13^ ß_2_-adrenergic receptor agonists (e.g., epinephrine),^14,15^ and nitric oxide (NO) donating moieties^16^ act directly at the JCT/inner wall endothelium, inhibiting cellular actomyosin contractility, weakening focal contacts (cell–extracellular matrix [ECM] adhesions), and degrading the actin microfilament network, overall relaxing the cells and the tissue as a whole.^14,16–19^ M3 muscarinic cholinergic receptor agonists (e.g., pilocarpine) stimulate ciliary muscle contraction, which passively expands the JCT and dilates SC.^20^ Prostaglandin FP-receptor agonists such as PGF_2α_ analogues upregulate matrix metalloproteinase synthesis and release in the ciliary muscle^21–23^ and are the most widely prescribed antiglaucoma agents. They work by enhancing aqueous humor outflow primarily via the unconventional uveoscleral outflow pathway from the anterior chamber through inner uveal meshwork, the iris root, the face of the ciliary muscle, and the spaces between the muscle bundles, and then exiting the eye through the sclera or the spaces around the scleral emissaria, with some fluid possibly being reabsorbed by the choroidal vasculature or lymphatics.^24–26^ Still other drugs such as ß_2_-adrenergic antagonists (e.g., timolol), α_2_-adrenergic agonists (e.g., brimonidine), and carbonic anhydrase inhibitors (e.g., acetazolamide, dorzolamide) inhibit aqueous humor formation.^26^

These drugs may all have systemic and/or local side effects, but as, or more importantly since POAG is a chronic disease, compliance issues have been a major concern,^27,28^ as the patient is in effect the delivery system at least once daily and often more frequently. Surgical treatments have their own complications such as cataracts, infection, hypotony, and acute loss of vision. A therapeutic agent with long-lasting efficacy and an improved safety profile would be a significant advance in glaucoma treatment.

Recently, the possibility of decreasing outflow resistance via gene therapy using TM- or SC-targeted transgene delivery systems has generated considerable interest.^29–39^ Several viral vectors based on adeno-associated virus (AAV), adenovirus (AdV), herpes simplex virus (HSV), and lentivirus have been used for gene delivery to the ocular anterior segment in animals, but lentiviral vectors have some advantages. One potentially significant advantage is their capability to integrate into postmitotic cells, which might be important for long-term transgene expression.^40^ Three types of lentiviral-based vectors have been studied, including human immunodeficiency virus type 1 (HIV-1), feline immunodeficiency virus (FIV), and equine infectious anemia virus (EIAV). FIV vectors were shown to have sustained transgene expression in the TM for more than 2 years in domestic cats^41^ and nonhuman primates,^30^ and in human anterior segment organ culture.^42,43^ However, expression of the transduced reporter gene, green fluorescent protein (GFP), seems to be variable in some studies even after equal numbers of transducing units were injected.^30,35^

Mammalian cells express several intrinsic species-specific factors that provide resistance to viral infection that could reduce the efficiency of transduction, and some of them are proteasome-dependent and intrinsic restriction negatively affects lentiviral-mediated gene delivery to stem, hematopoietic, and natural killer cells,^44–46^ but to date, the impact of intrinsic resistance factors on lentiviral transduction efficiency has not been explored in ocular tissues.

Our goal was to test the hypothesis that short-term inhibition of proteasome activity with MG132 would increase the efficiency of transduction of an FIV vector expressing mCherry fluorescent protein (mCherry) or green fluorescent protein (GFP) in immortalized human trabecular meshwork (TM-1) cells and monkey organ-cultured anterior segments (MOCAS). We also wished to determine if pretreatment with MG132 would allow for vector sparing by increasing the efficiency of gene delivery using lower doses of vector.

## Materials and Methods

### Cell Culture

HEK293TN and TM-1 cells were maintained in Dulbecco's modified Eagle's medium (DMEM) supplemented with 10% heat-inactivated fetal bovine serum (FBS), 100 μg/mL streptomycin, and 100 U/mL penicillin at 37°C in 5% CO_2_. TM-1 cells are SV-40 large T antigen-transformed primary human TM cells.^47,48^ MG132 (no. C2211; Sigma-Aldrich Corp., St. Louis, MO, USA) was dissolved in 0.5% dimethyl sulfoxide (DMSO) (no. D2560; Sigma-Aldrich Corp.) in DMEM. TM-1 cells were plated in a 24-well plate at a density of 1.5 × 10^4^ cells/well. Cells were pretreated for 1 hour with DMSO (0.5%) or 5, 10, 20, or 50 μM final concentrations of MG132 with a final adjusted DMSO concentration of 0.5%. Cells were then transduced with FIV.GFP at various multiplicities of transduction (MOT). After a 60-minute incubation, the media were replaced and cells were incubated for 2 days. Duplicate plates, one for imaging and one for DNA analysis, were established and treated at the same time. Note that whenever MG132 is mentioned in the text, 0.5% DMSO was also present.

### Toxicity Evaluation and Setting Baseline Multiplicity of Transduction in Cell Culture

MG132 cytotoxicity was determined using a commercially available assay Cell Titer 96 Aqueous solution–MTS assay (no. G3580; Promega, Madison, WI, USA). TM-1 cells were seeded in 96-well plates at 2 to 4 × 10^4^ cells/well and grown to confluence overnight in DMEM with 10% FBS at 37°C, 5% CO_2_. The next day, the media were aspirated and replaced with 100 μL media containing 50, 25, 12.5, 6.25, or 3.125 μM MG132 in DMSO. Control wells contained media only, media with 0.5% DMSO, or media with 0.5% IGEPAL CA-630 (no. I3021; Sigma-Aldrich Corp.). Note that the DMSO concentration in all wells was adjusted to 0.5%. The blank was set as a well with media only. The plate was incubated at 37°C, 5% CO_2_ for the three conditions tested: (1) 1-hour MG132 exposure and assayed immediately; (2) 1-hour exposure to MG132, followed by 3× PBS wash, then media replacement and assay 24 hours later; or (3) 24 hours of MG132 exposure. At the end of the incubation period, 20 μL Cell Titer 96 Aqueous solution was added to each well, and the plate was returned to the incubator for 1 hour. At that point, the reaction was stopped by adding 25 μL 10% sodium dodecyl sulfate (SDS) and absorbance measured at 490 nm using a Biotek Neo2 Plate Reader (Biotek, Winooski, VT, USA). Samples were run in either duplicate (24-hour incubation) or triplicate (1-hour incubation). To set a baseline transduction efficiency that allowed for the determination of increased transduction, we exposed TM-1 cells to FIV.GFP at various MOT in the absence of MG132, and 3 days later we determined the percentage of transduced cells by manual inspection.

### Packaging of Lentiviral Vectors

Packaging of the FIV vector was carried out as we have described previously.^49^ Briefly, the FIV-based vectors, pCDF1-MSC2-EF1-copGFP or pCDF1-CMV-mCherry--EF1-copGFP vector (1.74 mg; System Biosciences, Mountain View, CA, USA) were cotransfected into HEK293TN cells with pFIV-34-N (326 μg) and pVSVG (408 μg) in eight 500-cm^2^ culture plates (no. 431110; Corning, Corning, NY, USA). Culture supernatants were collected after 48 hours and centrifuged at 24,000*g* for 30 minutes to pellet viral particles. The pellets were resuspended in Hanks balanced salt solution (HBBS; Mediatech, Manassas, VA, USA) and centrifuged through a 20% sucrose cushion in phosphate-buffered saline (PBS). Viral pellets were then resuspended in HBBS, aliquoted, and stored at −80°C. Viral titers were determined using Crandell feline kidney cells (CrFK) and microscopically counting fluorescent cells following serial dilution. Stock viral titers were approximately 1 × 10^9^ transducing units (TU)/mL.

### Manual Quantification of Transduction Efficiency

TM-1 cells cultured on glass cover slips precoated with poly-L-lysine were treated with MG132 and then FIV.GFP as described above. Three days later TM-1 cells were washed two times with PBS and fixed in 4% paraformaldehyde in PBS. The cells were permeabilized with 0.5% Triton X-100. Cover slips were blocked by incubation in 5% FBS for 30 minutes followed by antibody staining. The primary and secondary antibodies were rabbit anti-copGFP (no. AB501, 1:1000 dilution; Evrogen, Moscow, Russia) and anti-rabbit Alexa Fluor 488 (no. A11008, 1:400 dilution; Life Technologies, Carlsbad, CA, USA), respectively, and were incubated for 1 hour each at 37°C . The nuclei were then labeled by incubating cover slips with 1 μg/mL Hoescht 33342 (no. H1399; Life Technologies) for 4 minutes at room temperature. Images were taken using a Zeiss Axioplan 2 microscope equipped with an Axiocam HRm camera using AxioVision 4.8 software (Carl Zeiss MicroImaging GmbH, Oberkochen, Germany). Nontransduced cells and cells transduced with FIV.GFP alone were used as controls, and the GFP expression was quantified by counting GFP-positive and -negative cells in five random fields at a magnification of ×40 so that at least 250 cells were counted for each sample. Quantification was done in a masked fashion.

### Quantification of Transduction by Flow Cytometry

The TM-1 cells were plated in a 12-well plate at a density of 2.5 × 10^5^ cells/well. Cells were pretreated for 1 hour with DMSO (0.5%, final concentration) or 5, 10, 15, 20, or 50 μM final concentrations of MG132 in 0.5% DMSO. Cells were then transduced with FIV.mCherry at a MOT of 20. After a 60-minute incubation, the media were replaced and cells were incubated for 2 days. On the third day, TM-1 cells were trypsinized and single-cell suspensions were made. The TM-1 cells for each sample were collected by centrifugation at 300*g*, 4°C for 5 minutes followed by resuspension of cell pellets in PBS containing 2% FBS.

To distinguish live from dead cells, 4′,6-diamidino-2-phenylindole (DAPI) (no. 62248; Thermo Scientific, Rockford, IL, USA), at a final concentration of 500 ng/mL, was added to the single-cell suspensions and incubated for 1 minute with gentle mixing followed by analysis in the flow cytometer. Samples were analyzed on a BD LSRII flow cytometer (Beckton Dickinson, San Jose, CA, USA) running Diva v8.0. mCherry was excited using a 150-mW 561-nm laser and detected with a 610/20-nm bandpass filter behind a 600-nm long-pass dichroic filter. The data were analyzed using FlowJo version 10.0.8r1 (FlowJo LLC, Ashland, OR, USA). Briefly, cells were gated based on morphology in a plot of forward scatter versus side scatter. Doublets were eliminated using forward scatter area versus forward scatter height, and dead cells were excluded based on DAPI fluorescence. The final population of live, single cells was assessed for mCherry fluorescence with the gate set based on the untransfected control sample.

### Monkey Organ-Cultured Anterior Segments

Eyes were obtained from 14 rhesus (*Macaca mulatta*) monkeys that were euthanized for nonocular studies at the Wisconsin National Primate Research Center. MOCAS were established according to Hu et al.^50^ Following 2 days of equilibration at 2.5 μL/min, baseline outflow facility was measured by two-level constant pressure perfusion.^51^ Fluid flow from an external reservoir was measured for approximately 1 hour, alternating every 4 minutes between two pressures 10 mm Hg apart. Outflow facility was calculated as the change in flow divided by the change in pressure, and was sequentially averaged. Anterior segment pressure in MOCAS was monitored (Isotech transducers/HSE Tam-D amplifier; Harvard Apparatus, Holliston, MA, USA) and recorded every 15 minutes throughout the experiment, except when outflow facility was being measured or to replenish medium in the infusion syringe.

Paired anterior segments were injected through the inflow port with a 10-μL bolus injection of MG132 in DMSO to one segment or DMSO only to the other segment such that the final concentration in the anterior segment was 20 μM MG132 and/or 0.5% DMSO. After 1 hour, during which time the infusion pumps were turned off, both segments were then injected with a 10- to 20-μL bolus of FIV.GFP, either 0.8 × 10^7^ TU (three pairs) or 2 × 10^7^ TU (three pairs). Media infusion was then resumed. IOP was monitored continuously. Posttreatment outflow facility was measured on day 3 post treatment. In other cases where only a single eye was available, MOCAS were treated with FIV.GFP only (2 × 10^7^ TU, *n* = 7; 0.8 × 10^7^, *n* = 1; no DMSO and no MG132). We have previously established that DMSO at this concentration in live monkeys does not affect outflow facility.^52^ All studies were conducted in accordance with the ARVO Statement for the Use of Animals in Ophthalmic and Vision Research.

### Imaging MOCAS Tissues

Anterior segments were divided into four equal pieces. One segment was imaged (×5 magnification) with a Zeiss AxioVert 200M inverted fluorescence motorized microscope (Carl Zeiss MicroImaging GmbH) to determine the distribution of GFP expression in the tissue. For quantification of GFP expression, nonoverlapping GFP images covering 95% of each monkey eye segment were converted to JPG files using AxioVision Rel. 4.8 software (Carl Zeiss MicroImaging GmbH). The total GFP density in each image was then measured by ImageJ software as described previously.^53^ The length of TM in each image was measured using AxioVision Rel. 4.8. Then the total GFP density and the total TM length were obtained by adding up the data from the GFP images for each monkey eye segment. The GFP intensity for each sample was normalized by TM length, and the GFP intensity of MG132-pretreated and FIV.copGFP-transduced monkey eyes was calculated by normalization to GFP intensity of their corresponding controls (DMSO-pretreated and FIV.copGFP-transduced monkey eyes).

To more precisely localize GFP expression in transduced MOCAS eye tissues, a second segment was fixed with 4% paraformaldehyde and embedded in paraffin. For histologic evaluation, 5-μm paraffin sections were cut and processed according to standard protocols. Sections were analyzed under a fluorescence microscope equipped with appropriate filters (Carl Zeiss MicroImaging GmbH, Axio Imager) to detect GFP fluorescence or for regular light microscopy. For detection of α-smooth muscle actin (α-sm-actin), sections were initially treated with boiling citrate buffer (1 × 30 minutes, pH 6), followed by two washing steps in H_2_O and 1× Tris-buffered saline (TBS, 10 mM Tris pH 7.0, 0.15 M NaCl). Sections were then blocked with 2% BSA, 0.2% cold water fish gelatin (Aurion, Wageningen, The Netherlands), all in 1× TBS for 1 hour at room temperature. After blocking, sections were incubated for 1 hour at room temperature with rabbit polyclonal anti-α-sm-actin IgG (1:75 dilution; GeneTex, Irvine, CA, USA). After three washes with TBS, sections were incubated with biotinylated secondary antibodies (Vector Laboratories, Burlingame, CA, USA) diluted by 1:500 for 1 hour at room temperature, and afterward incubated with Streptavidin Alexa 555 (Molecular Probes, Eugene, OR, USA) diluted by 1:1000 for 1 hour at room temperature in the dark. Sections were washed again three times and counterstained with DAPI (no. H-1500, Vectashield; Vector Laboratories) diluted in 1:10 fluorescent mounting medium (Dako, Glostrup, Denmark).

CD31 is a marker for vascular endothelial cells that is known for its robust labeling of SC endothelial cells.^54^ For CD31 staining, sections were washed in 0.1 M phosphate buffer for 5 minutes and then blocked with 5% nonfat dry milk in 0.1 M phosphate buffer for 1 hour at room temperature. After blocking, sections were incubated with goat anti-CD31 (1:50 dilution; R&D Systems, Wiesbaden, Germany) overnight at 4°C. Donkey anti-goat IgG (1:2000) conjugated to Cy3 (Jackson ImmunoResearch, West Grove, PA, USA) was used as secondary antibody. Sections were washed again three times with phosphate buffer and counterstained with DAPI (Vectashield) diluted in 1:10 fluorescent mounting medium (Dako). The sections were analyzed under a fluorescence microscope (Carl Zeiss MicroImaging GmbH, Axio Imager). For negative controls, primary antibodies were omitted and the sections were incubated with secondary antibodies only. For GFP immunoreactivity, sections (5 μm) were cut, mounted on glass slides, and stored at −20°C until they were processed for immunofluorescence microscopy. Sections were warmed to room temperature, deparaffinized, and then blocked as described above. Sections were then incubated with 1:10,000 dilution of rabbit anti-copGFP antibody (Evrogen) and washed in PBS, followed by a 1:400 dilution of anti-rabbit Alexa Fluor 488 (Life Technologies) for 1 hour each at 37°C, respectively. The nuclei were labeled by incubating sections with 1 μg/mL Hoechst 33342 (Life Technologies) for 4 minutes at room temperature.

### Genomic DNA Isolation

DNA was extracted from TM-1 cells using the FlexiGene DNA Kit (no. 51204; Qiagen, Valencia, CA, USA) per the manufacturer's protocol. For MOCAS samples, frozen monkey eye segments (full quadrants) were homogenized in 300 μL ice-cold extraction buffer (NaCl 10 mM, Tris-HCl 10 mM pH7.5, EDTA 10 mM, 100 μL SDS 5%, 15 μL proteinase K) using a Bullet Blender 24 (Next Advance, Averill Park, NY, USA) and incubated in a water bath at 60°C for 30 minutes. The homogenates were centrifuged at 21,130 *g* at 4°C for 15 minutes to pellet debris. A total of 200 μL supernatant from each sample was transferred to a clean 1.5-mL microcentrifuge tube, and 200 μL 5 M ammonium acetate and 400 μL isopropanol were added. The samples were mixed by inverting the tubes five times, and incubated at room temperature for 10 minutes. The samples were then centrifuged at 21,130 *g* at 4°C for 5 minutes using an Eppendorf model 5424 microcentrifuge (Eppendorf, Hamburg, Germany). The pellets were washed with 500 μL 70% ethanol and centrifuged again at 21,130 *g* at 4°C for 5 minutes. The DNA pellets were air-dried at room temperature for 10 to 15 minutes and were resuspended in 50 μL Tris-EDTA (20 mM Tris, 1 mM EDTA, pH 7.4) with RNase. The quality and quantity of DNA were determined using a Nanodrop Lite Spectrophotometer (Thermo Scientific, Waltham, MA, USA).

### Quantitative PCR for Vector Genome Equivalents

Quantitative real-time PCR was carried out following the manufacturer's protocol for RT^2^ SYBR Green using an Applied Biosystems 7300 Real Time PCR System (Foster City, CA, USA). Rox qPCR Mastermix (no. 330520; Qiagen) was used. The β-actin gene was used as the reference gene. For TM-1 cells the human β-actin sequence (Genbank no. NM_001033084) was used for designing the primers. For the MOCAS samples the *M. mulatta* β-actin gene (GenBank no. XM_006715764) was used. The primer sequences were as follows: (1) copGFP sense primer, 5′-CTTCCCGTATGGCTTTCATT-3′; (2) copGFP antisense primer, 5′-GTTGCGTCAGCAAACACAGT-3′; (3) monkey-β-actin sense primer, 5′-TCCTCCCTGGAGAAGAGCTA-3′; (4) monkey-β-actin antisense primer, 5′-AAGGTTGGAAGAGAGCCTCA-3′; (5) human-β-actin sense primer, 5′-CTCTGACCTGAGTCTCCTTT-3′; (6) human-β-actin antisense primer, 5′-CACCCACAACACTGTCTTAG-3′; (7) mCherry sense primer, 5′-CCGTAATGCAGAAGAAGACCA-3′; (8) mCherry antisense primer, 5′-CTTCAGCCTCTGCTTGATCTC-3′ The standards were made by serially diluting (10-fold) the pCDF1-copGFP or the pCDF1-mCherry vector plasmid vectors. The β-actin standards were made by serially diluting (10-fold) the pooled MOCAS DNA samples or pooled TM-1 DNA samples. Data analysis was carried out as described by Hoebeeck et al.^55^ Because it was not feasible to determine the actual number of cells transduced in MOCAS, we chose to present the quantitative (q)PCR data as vector genomes normative to actin, for both the cell culture studies and the MOCAS studies, so the data do not represent vector genome equivalents per cell. Quantifying viral genome equivalents by measuring vector DNA is a more accurate measure of the delivery of vector genomes than RT-qPCR for GFP mRNA because there is no amplification step from transcription of the vector genomes.

### Statistical Analyses

GFP immunofluorescence in cells and MOCAS was compared using the Mann-Whitney *U* test. The effect of MG132 on aqueous outflow facility, quantification of GFP expression by ImageJ (http://imagej.nih.gov/ij/; provided in the public domain by the National Institutes of Health, Bethesda, MD, USA), and quantification PCR of copGFP or mCherry gene was assessed using the paired *t*-test. All statistical analyses were performed using the Statistical Package for the Social Sciences, version 16.0 (SPSS, Chicago, IL, USA). Statistical significance was defined as a *P* value < 0.05.

## Results

### Toxicity Evaluation and Baseline Transduction

**Figure 1:**
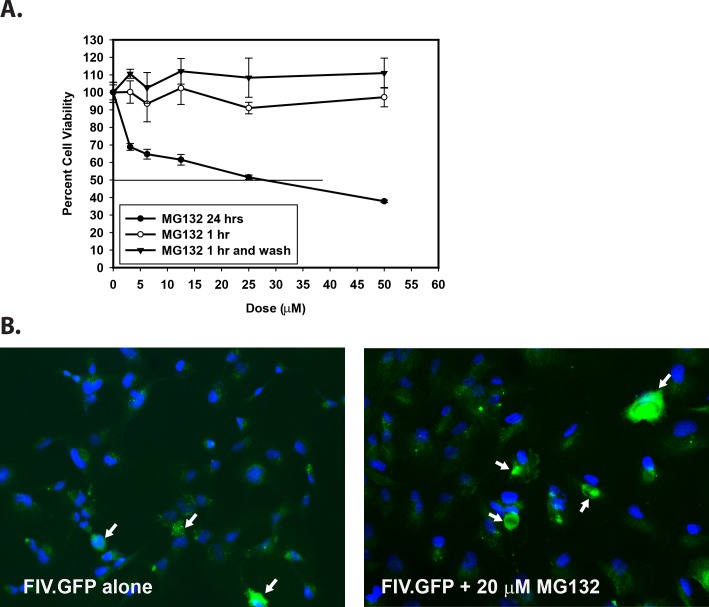
Toxicity of MG132 in TM-1 cells. (A) TM-1 cells were incubated with various concentrations of MG132 in the presence of 0.5% DMSO for 1 hour followed directly by assay; 1 hour with PBS wash, replacement by media, and assay 24 hours later; or 24 hours; and toxicity was evaluated using the MTS assay. Toxicity was not evident in the cells exposed to MG132 for 1 hour and then assayed or treated for 1 hour followed by assay 24 hours later, up to concentrations of 50 μM, so a CC_50_ value could not be calculated. MG132 was toxic after 24 hours of exposure with a CC_50_ value of 28 μM. DMSO at 0.5% was not toxic (97% viability at 1 hour, 103% viability at 1 hour and wash, and 90% viability at 24 hours). The experiments were done two or three times and the results were averaged. (B) Representative images of cell cultures used to determine the effect of MG132 treatment on transduction efficiency. Note that TM-1 cells have a low level of autofluorescence in the GFP channel in the absence of the FIV.GFP vector, so only bright cells (denoted by arrows) were counted.

Prior to testing the effect of MG132 on transduction efficiency of TM-1 cells, we first determined MG132 toxicity. TM-1 cells were exposed to various concentrations of MG132 in the presence of 0.5% DMSO (solvent) for either 1 hour, 1 hour with media replacement and assay 24 hours later, or 24-hour exposure to MG132, and toxicity was evaluated using a commercially available MTS assay. As shown in Figure 1A, an 1-hour exposure followed immediately by assay, or 1-hour exposure with MG132 washout and assay 24 hours later, resulted in no evidence of toxicity to the cells while exposure for 24 hours resulted in toxicity with a CC_50_ value of 28 μM. To further evaluate toxicity, TM-1 cells were exposed to various concentrations of DMSO and MG132 and cytotoxicity was evaluated by determining the percentage of rounded cells. With regard to morphologic changes in cells, concentrations above 0.5% DMSO and 50 μM MG132 were toxic and resulted in cell rounding and sloughing from the plates (data not shown). Therefore, for all subsequent studies the maximum concentrations used were 0.5% DMSO and 50 μM MG132.

We determined that transduction of TM-1 cells with 20 transducing units of FIV.GFP per cell in the absence of MG132 resulted in transduction of approximately 20% of the cells, and this was set as the baseline for testing the effect of MG132. Examples of the transduction experiments are shown in Figure 1B.

### Proteasome Inhibition Increased Transduction of Cultured TM-1 Cells

**Figure 2:**
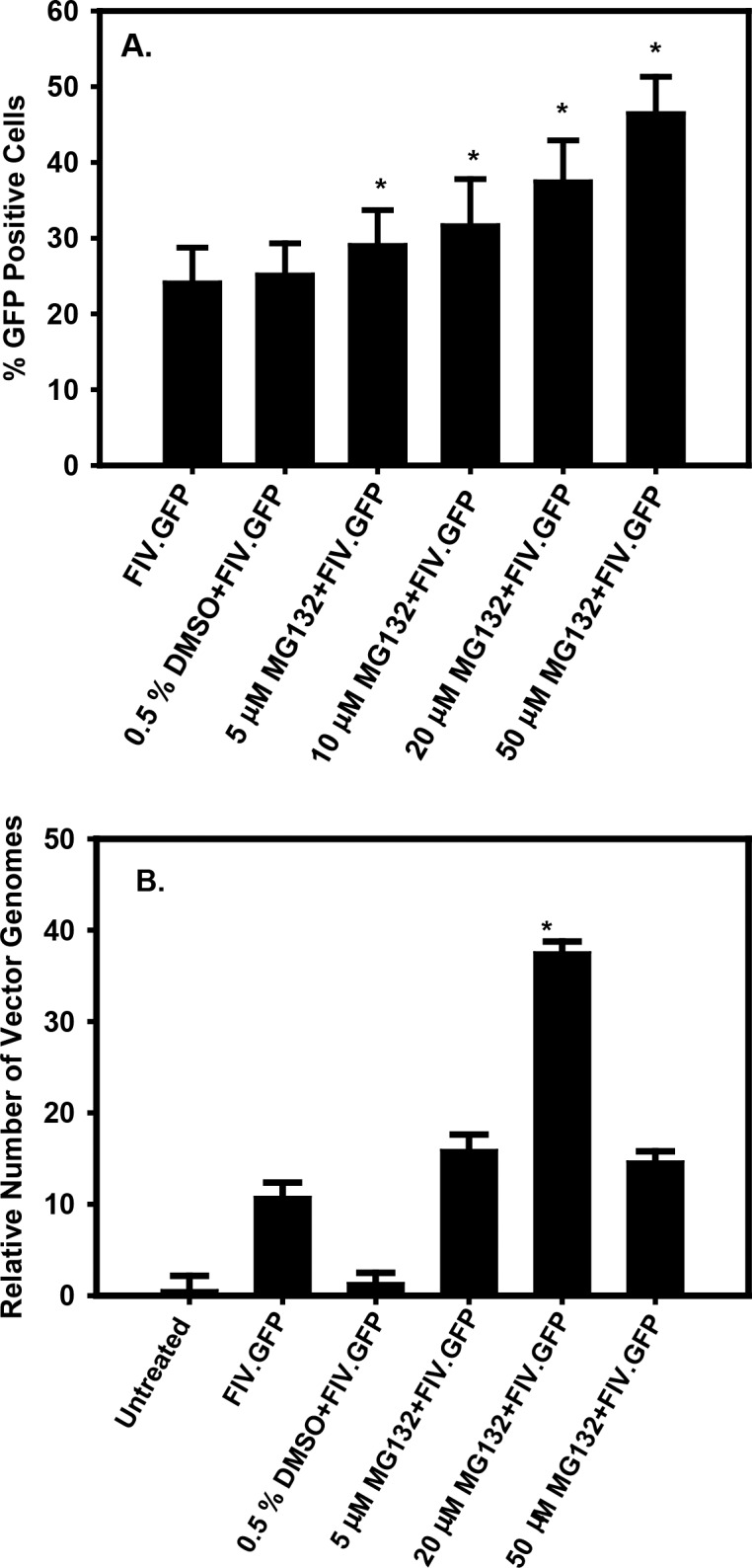
Quantification of TM-1 cell transduction by microscopy and qPCR. TM-1 cells were left untreated, pretreated with MG132 solvent only, or pretreated with various concentrations of MG132 for 1 hour followed by transduction with FIV.GFP. The final DMSO concentration was adjusted to 0.5% in all samples. (A) The percentage of GFP+ cells as determined by counting the cells in five random microscope fields at a magnification of ×40. A minimum of 250 total cells were counted for each condition in a masked fashion and the experiment was done twice. (B) qPCR of total cellular DNA isolated from parallel cultures with primers for copGFP normalized to β-actin. Note that the assay measures both integrated and episomal vector DNA. Asterisks indicate P < 0.05 compared to the FIV.GFP sample only. The experiment was done twice and the results were averaged.

To determine if proteasome inhibition increased transduction efficiency, TM-1 cells were treated with increasing concentrations of MG132 for 1 hour, rinsed with culture medium, and then exposed to the FIV.GFP vector at a MOT of 20. A total of 250 cells from five random microscope fields at ×40 magnification were counted for each sample. There was a speckled pattern of FIV.GFP expression in human TM-1 cells, and the number of cells that were GFP positive increased as the concentration of MG132 was increased up to 50 μM. Figure 2A shows the percentage of cells that were GFP positive. In cultures treated with FIV.GFP only, approximately 24% of the cells were transduced and 0.5% DMSO did not significantly increase the percentage (*P* = 0.4). The number of transduced cells increased to 29% when 5 μM MG132 was included and the percentage continued to increase as the MG132 concentration was increased, with 46% of the cells being transduced at 50 μM MG132. The percentage of GFP-positive cells was significantly different from the FIV.GFP-only control at all MG132 concentrations (*P* < 0.05).

**Figure 3:**
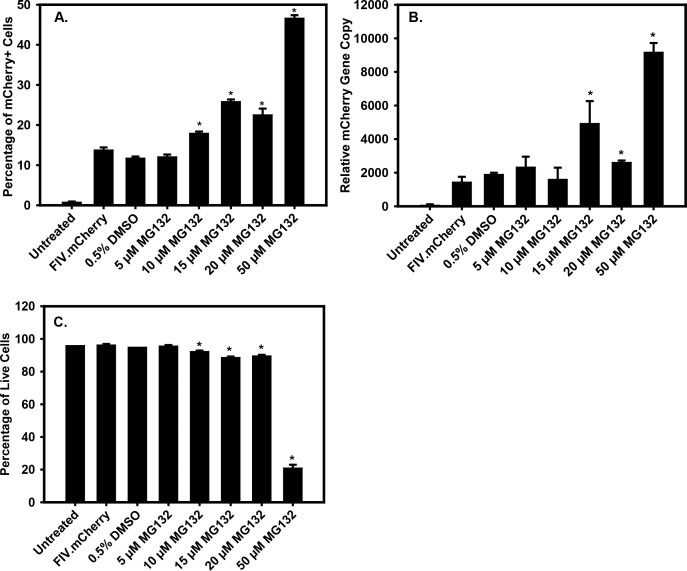
Quantitative analysis of FIV.mCherry transduction efficiency in TM-1 cells using flow cytometry. (A) The percentage of mCherry-positive cells in control and MG132-treated TM-1 cells. (B) Quantitative PCR determination of FIV.mCherry vector DNA normalized to β-actin in control and MG132-treated TM-1 cells. Note that this does not represent vector genomes per cell. (C) The percentage of cells that were alive in control and MG132-treated TM-1 cells as determined by DAPI staining. Asterisks indicate significantly different from the FIV.mCherry-only sample (*P < 0.05). All studies were done twice and the results were averaged.

To further quantify the increased transduction efficiency, we isolated DNA from the transduced cells and used a qPCR assay to determine the amount of vector DNA normalized to the cellular actin gene (Fig. 2B). In FIV.GFP-transduced cells without MG132 treatment, there were approximately 10 vector genome equivalents. Treatment with DMSO (solvent control) alone decreased the number of genomes to nearly baseline. There were approximately 15 vector equivalents in cells pretreated with 5 μM MG132 and this increased to nearly 40 equivalents in cells pretreated with 20 μM MG132. Treatment with 50 μM MG132 reduced the number of vector equivalents to approximately 15. The reduced number of vector genomes in the 50 μM samples is most likely due to toxicity of MG132 even though the morphology of the cells appeared normal (see Fig. 3C). Note that these numbers do not represent vector genome copies per cell, but rather the amount of vector DNA normalized to cellular actin.

### Flow Cytometry Analysis of MG132-Mediated Enhancement of Transduction

To confirm the results from microscopically counting GFP-positive cells, we also determined the percentage of transduced cells using flow cytometry. For these experiments, we first noted that the background autofluorescence of TM-1 cells was such that we could not set the gating sufficiently to quantify the GFP-positive cells. Therefore, for these studies we used a mCherry-expressing FIV vector.

Representative examples of the flow cytometry data showing the gating parameters are shown in Supplementary Figure S1. Quantitative results are shown in Figure 3A. For the mCherry.FIV-only samples, approximately 13% of the cells were transduced. Similar results were seen in the DMSO (solvent)-only control and the cells treated with 5 μM MG132. The percentage of transduced cells increased to 20% in the 10 μM-treated samples and increased to 27% in the 15 μM sample. In the 20 μM sample, 24% of the cells were transduced and in the 50 μM-treated sample, 47% of the cells were transduced. The 10, 15, 20, and 50 μM treatment groups were all significantly different from the mCherry.FIV-only group (*P* < 0.05). It should be noted that the 50 μM treatment was cytotoxic (see Fig. 3C) and that only living cells were counted in each sample, so it is likely that the high percentage of total transduced cells in that sample may be skewed as a result of the counting procedure.

To confirm the counting results, we also determined the amount of vector DNA normalized to cellular actin DNA in the sorted positive cells using qPCR (Fig. 3B). Vector equivalents were similar in the mCherry.FIV-only samples and the DMSO 5 and 10 μM treatment groups. Vector genome equivalents were significantly increased (*P* < 0.05) compared to mCherry.FIV in the 15, 20, and 50 μM groups. As noted above, the amount of vector genome equivalents in the 50 μM group may be skewed higher due to the sorting procedure, which eliminated dead cells.

Figure 3C shows the percentages of live cells for each of the groups as an indicator of toxicity. Toxicity was not observed for the untreated, mCherry.FIV, DMSO, and 5 μM MG132 groups. Slight toxicity was seen in the 10, 15, and 20 μM groups (significantly different from the mCherry.FIV group, *P* < 0.05), but the 50 μM treatment was highly toxic, with only 20% of the cells in the sample being viable.

### Effect of MG132 on FIV.GFP Expression in MOCAS

**Figure 4:**
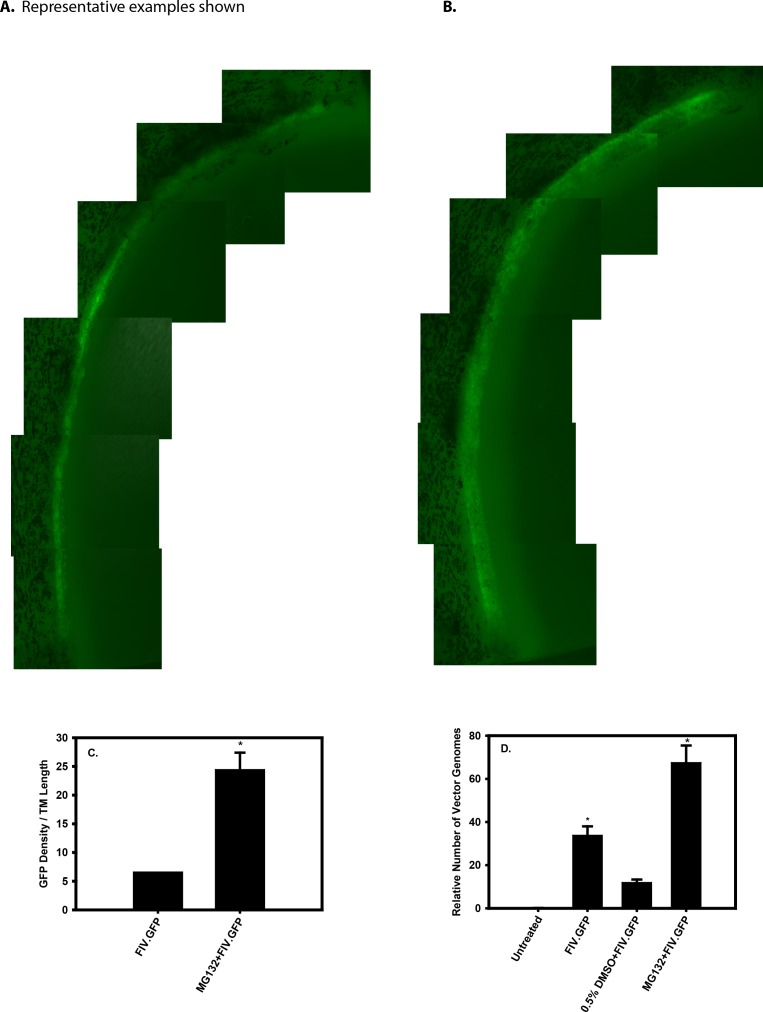
FIV.GFP transduction in the trabecular meshwork of MOCAS. (A, B) Representative examples of GFP expression in FIV.GFP-transduced trabecular meshwork of MOCAS that were used for quantification of GFP signal. (A) Control eye, 0.5% DMSO + FIV.GFP; (B) 20 μM MG132 + 0.5% DMSO + FIV.GFP. Note the wider expression band in the eye treated with 20 μM MG132 (B) compared to control eye (A) that was treated only with 0.5% DMSO and FIV.GFP. Images (×20) were taken with a Zeiss AxioVert 200M inverted fluorescence microscope. (C) Quantification of GFP density in the trabecular meshwork of MOCAS determined using ImageJ and AxioVision Rel. 4.8. (D) Quantitative PCR determination of GFP vector DNA normalized to β-actin in transduced TM segments. Asterisks indicate significantly different (P < 0.05) from control (FIV.GFP + DMSO) or baseline (FIV.GFP only).

We determined if proteasome inhibition increased transduction efficiency in our physiologically relevant MOCAS model. Cultured anterior segments were pretreated with 20 μM MG132 for 1 hour and were then transduced with either a high or a low dose, 2 × 10^7^ or 0.8 × 10^7^ TU, respectively, of FIV.GFP vector. We used FIV.GFP in these studies because our imaging system is set to detect GFP and not mCherry. The tissues were harvested 3 days post vector delivery and then analyzed by fluorescent imaging and qPCR. GFP expression was detected in the control eye transduced with a high dose of vector in the absence of MG132 (Fig. 4A). We saw a thin band of GFP signal corresponding to the location of the TM, indicating transduction of the tissue, and noted that there were areas where portions of the TM were not transduced. A representative image from an eye treated with 20 μM MG132 is shown in Figure 4B. The GFP signal was more intense and the band of tissue that was GFP positive was wider than in the control eyes (Fig. 4A). In addition, there were fewer areas of GFP signal dropout in MOCAS treated with MG132.

To quantify the amount of GFP expression in MOCAS, the fluorescence images were converted to JPEG files and the GFP signal was quantified using ImageJ. Figures 4A and 4B show examples of the images that were used for quantification. Nonoverlapping images covering each quadrant were quantified, normalized by dividing by the length of the TM in the quadrant of tissue, and then averaged. The GFP density divided by the TM length data is shown in Figure 4C. There was an approximately 5-fold increase in GFP density in the MG132 + FIV.GFP-treated MOCAS than in the FIV.GFP-only MOCAS (*P* < 0.05), indicating that MG132 treatment significantly increased the transduction efficiency.

To further quantify the transduction efficiency, we isolated DNA from one tissue segment from each eye and determined the amount of vector DNA normalized to cellular β-actin. The results are shown in Figure 4D. There was no GFP signal in the nontransduced control tissue. We found approximately 35 vector equivalents in the FIV.GFP-only group (no MG132 and no DMSO). This was reduced to approximately 10 vector equivalents in the eye that was treated with FIV.GFP vector and 0.5% DMSO. In the eye treated with MG132 and FIV.GFP, we found approximately 70 vector equivalents, which was significantly different from the FIV.GFP-only sample (*P* < 0.05).

### Immunostaining and Histology in MOCAS

**Figure 5:**
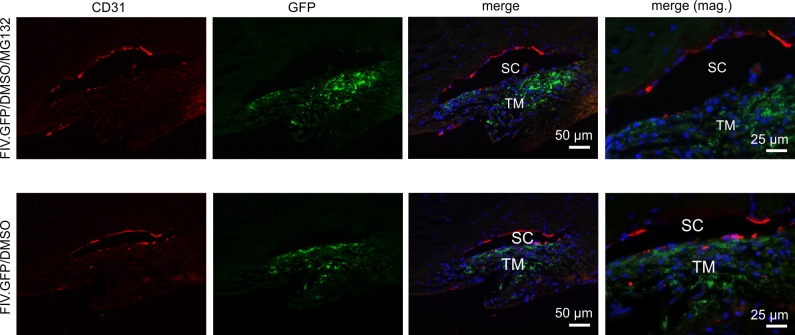
Localization of lentiviral-mediated transduction. MOCAS were treated with 20 μM MG132 for 1 hour, then transduced with 2 × 10^7^ units of FIV.GFP. Sections were stained with antibody to copGFP (Alexa 488 fluorophore) to localize transduced cells and anti-CD31 antibody (Cy3 fluorophore) to identify Schlemm's canal cells. Nuclei (blue) were stained with DAPI. Note that transduction occurred only in the TM.

The histology of the TM outflow pathways of each MOCAS quadrant was analyzed in all eyes. In hematoxylin- and eosin-stained paraffin sections, the overall structure of TM and SC was intact (Supplementary Fig. S2). Numerous cells covered the trabecular beams and surrounded the lumen of SC as a largely continuous endothelial layer. At the very posterior aspect of the TM, cells of the anterior tips of the longitudinal portion of the ciliary muscle were present, indicating the position of the cut by which the rest of the ciliary body had been removed during processing of the anterior eye segments for organ culture (Supplementary Fig. S2). The remaining muscle tips were usually not longer than approximately 10 μm, although in some quadrants, longer muscle tips with a length of up to 100 μm were observed. Between the muscle cells, pigmented melanocytes were occasionally present, a characteristic feature of the monkey ciliary muscle.^56^ The identity of the remaining muscle cells was confirmed by immunohistochemistry with antibodies against a-SMA (Supplementary Fig. S2). The anterior tips of the ciliary muscle were always rotated anterior-outwardly, thereby covering the posterior TM at various lengths. We attribute this rotation to the elasticity of the anterior tendons of the monkey ciliary muscle^56,57^ that contract when the posterior attachment of the ciliary muscle is cut.^58,59^ Remnants of iris or ciliary processes were not observed. To confirm gene delivery to the TM cells, we analyzed GFP fluorescence in paraffin sections (Fig. 5). GFP fluorescence was barely detectable in the TM of samples treated with FIV.GFP (0.8 × 10^7^ TU, data not shown) and more robust in samples treated with 2 × 10^7^ TU FIV.GFP. There was an apparent increase in GFP staining in samples treated with 2 × 10^7^ TU FIV.GFP and 20 μM MG132. Note that this GFP staining assay is nonquantitative and only indicates localization of the signal. No GFP fluorescence was detectable in samples that had not been treated with virus. Overall, there was little, if any, direct GFP reactivity or GFP immunostaining in sclera, corneal endothelium, or anterior ciliary muscle remnants (not shown). To clarify whether SC endothelial cells had been transduced by virus, we combined CD31 immunohistochemistry with direct detection of GFP fluorescence. CD31 staining, a marker known for its robust labeling of SC endothelial cells,^54^ was observed in the endothelial cells of the outer wall of SC (Fig. 5). In the inner wall endothelium, some gaps were present, which we attribute to focal loss of SC endothelial cells.^60^ We did not detect any overlap between CD31 immunoreactivity and GFP fluorescence, indicating that SC cells had not been transduced by virus (Fig. 5).

### Outflow Facility

To determine if MG132 treatment altered physiological parameters, we measured outflow facility in MOCAS. The mean + SEM ratio of the treated to baseline outflow facility was 1.315 ± 0.395 for the 20 μM MG132/0.5% DMSO + FIV.GFP eyes and 1.121 ± 0.150 for the control FIV.GFP ± DMSO eyes. Neither ratio differed significantly (*P* > 0.05) from the no-effect ratio of 1.0, both ratios were within the expected resistance washout effect seen during anterior chamber perfusion in MOCAS^33^ or live monkey eyes,^61^ and the ∼20% difference between the groups was neither statistically significant nor clinically meaningful (data not shown).^62^

## Discussion

Significant problems with the current therapeutic approach for glaucoma are patient compliance, requiring long-term self-administration of drugs due to the chronicity of the disease, and the occurrence of local and occasionally systemic side effects. Alternative therapeutic approaches that require less frequent administration could significantly improve glaucoma treatment. One such approach is gene delivery where long-term expression of a gene that increases outflow facility could reduce the number of treatments needed and reduce the impact of patient noncompliance.

Several different viral gene delivery vectors have been evaluated for use in treating glaucoma including AdV,^32^ AAV,^31,39,63,64^ HSV,^65^ and FIV^30^ viral vectors. A number of problems with the first three vectors have been identified, including short-term transgene expression with AdV vectors, difficulties with transducing TM cells with standard AAV vectors, and difficulties in producing very high titer HSV vector stocks. AAV-mediated delivery to the TM has been shown for self-complementary AAV vectors and capsid tyrosine-modified AAV vectors^66^ but the carrying capacity of such vectors is severely limited. Lentiviral vectors (FIV, HIV-1, and EIAV) have some advantages, including the ability to permanently integrate in nondividing cells and larger carrying capacity than AAV, although insertional mutagenesis is a potential disadvantage of lentiviral vectors.^38^

Proteasome-mediated inhibition of viral infection clearly negatively affects the transduction efficiency of viral vectors. Adeno-associated vector transduction was increased by proteasome inhibition with tripeptidyl aldehydes (e.g., N-acetyl-L-leucyl-L-leucyl-L-norleucyl [LLnL] and MG132) and anthracycline derivatives (e.g., doxorubicin and aclarubicin) in human airway epithelial cells.^67^ Tang et al.^68^ demonstrated that proteasome-modulating agents increased rAAV-2–mediated transgene expression in human intestinal epithelial cells. The proteasome-mediated inhibition of AAV vector transduction involves EGFR-PTK–mediated phosphorylation of surface-exposed tyrosine residues on the capsid.^69–72^ Tyrosine phosphorylated AAV capsids are then ubiquitinated and the capsids traffic to proteasomes where they are destroyed.^73^ In an effort to increase transduction efficiency, Zhong et al.^74^ used site-directed mutagenesis to remove the tyrosines from the AAV2 capsid and showed that gene delivery was enhanced with the mutant vectors. Modification of capsid surface lysine residues also increased transduction efficiency of AAV2 vectors.^75^ Tyrosine-modified AAV vectors have been used in clinical trials^76^ and efficiently deliver genes to the retina and TM in rodents. Thus, proteasome inhibition or avoidance is one potential way to increase transduction efficiency with viral vectors.^66,77^

One of the major problems with using lentiviral vectors is the presence of intrinsic host cell proteins that inhibit cellular infection.^78–83^ These restriction factors protect host cells from infection by different retroviruses, act by different mechanisms, play a role in the species specificity of these viruses, and reduce the efficiency of transduction. This is particularly important for ocular gene delivery due to the small volume of vector preparation that can be delivered. Currently, the specific proteasome-mediated lentiviral restriction factors in TM cells are not known and will require additional experiments. Given that the transduction efficiency of AAV vectors can be increased by preventing proteasome-mediated inhibition, and that Leuci et al.^44^ showed that transient proteasome inhibition increased the transduction efficiency of lentiviral vectors in CD34^+^ cells and T-cells, we reasoned that transient inhibition of proteasomes might increase FIV-mediated transduction of TM. In the current work, we showed that transient proteasome inhibition does significantly increase the transduction efficiency in TM cells in culture and in the TM in our monkey eye organ culture system. The inclusion of short-term proteasome inhibition could be a useful adjunct to viral gene delivery for treating glaucoma and may improve gene delivery in other cell types or tissues.

We noted two effects of MG132 treatment in the MOCAS. First, the width of the GFP expression band in the TM was increased, suggesting increased transduction efficiency that was confirmed using a quantitative DNA PCR assay to measure the amount of vector DNA in the tissue. Note that this assay measures both integrated and episomal viral DNA. The data indicated that there was a 2-fold increase in vector DNA in the MG132-treated eyes compared to FIV.GFP-transduced eyes. Second, we noted that the GFP signal was more uniform around the circumference of the TM, suggesting that more even transduction of the TM was obtained. We noted no effect of FIV.GFP, MG132/DMSO, or the combination thereof on outflow facility at the titers/doses used, and none was expected. The study was directed only toward the question of whether proteasome inhibition with MG132 would enhance GFP transduction in these tissues, as it indeed did.

There was an apparent discrepancy between the percentage of GFP-positive TM-1 cells exposed to 50 μM MG132 and the vector DNA present in these cells when we used cell counting to quantify transduction efficiency. The most likely explanation is that, although we did not see morphologic changes as an indication of toxicity in the cells exposed to 50 μM MG132, this treatment was toxic. The flow cytometry data support this explanation as only 20% of the cells in the 50 μM samples were classified as living based on DAPI staining. The quantification of vector DNA in the flow samples was done only with the living fraction. We also noted that DMSO alone significantly reduced transduction in the vector genome quantification assay. Based on our qPCR data, it is unlikely that DMSO alone is acting to increase transduction efficiency, suggesting that the effect is solely due to MG132 treatment.

Histologic examination of the MOCAS tissue revealed no evidence of toxicity or loss of cellularity in the TM. Some focal loss of SC inner wall endothelial cells was observed by CD31 immunostaining. Occasional gaps in this lining are not an uncommon finding in anterior segment organ cultures and were reported previously in the original paper describing this technique.^60^ Proteasome inhibition can be toxic in cell culture^84^ but that may be dependent on the concentration of inhibitor and the length of time the inhibition is maintained, as we found in the cell culture studies at 50 μM and higher concentrations. We found little to no toxicity in cells transiently exposed to MG132 for 1 hour using an MTS assay. Collectively these findings suggest that MG132 as administered here was not toxic to TM or SC cells, and that any effect on IOP would not be due to such a toxic response. While unexpected toxicity might be revealed using higher-resolution methods than employed here, the fact that a proteasome inhibitor, Velcade, has been approved by the Food and Drug Administration for clinical use in treating multiple myeloma^85^ also suggests that toxicity will not be an issue. Our data showing that short-term exposure of TM-1 cells to MG132 was not toxic supports this conclusion. Although we did not wash out the MG132 in the MOCAS experiments, it would have been rapidly diluted once fluid flow was resumed. It may be possible to increase the MG132 concentration in the MOCAS without causing toxicity because of the short-term nature of the exposure, and it would be interesting to determine if gene delivery is further increased under such conditions.

One issue with the use of lentiviral vectors for ocular gene delivery is that it requires the use of very high titer stocks. In order to prepare vector, it is necessary to concentrate the vector preparations several-fold, leading to several downstream effects. First, the potential for aggregation of vector particles increases and makes it difficult to deliver large numbers of particles. It also increases the expense of vector preparation. Thus, an additional goal of our study was to determine if proteasome inhibition could result in “vector sparing,” analogous to antigen sparing in vaccines, and as has been shown with AAV vectors.^74^ We reasoned that if we could increase the transduction efficiency we could use less viral vector to achieve the same therapeutic effect. We tested two different doses of vector, but found that MG132 treatment did not improve the transduction efficiency with lower doses of vector. Our results are consistent with those of other studies using FIV-mediated delivery in that there does not appear to be a dose-dependent response with increasing amounts of vector.^30,38,42^ Rather, it appears that there is a threshold effect and that a minimum number of vector particles are required to achieve any delivery. Whether there is a dosage effect above this minimum threshold is unknown, and will require additional work with more and higher doses, which might not be achievable in terms of stock vector titers that can be generated.

It would be of interest to determine which lentiviral intrinsic resistance proteins that have been identified in some cell types^78–83^ act to reduce transduction efficiency in TM cells. If so, then inhibiting multiple proteins might lead to even more efficient gene delivery.

In summary, we have shown that short-term exposure of TM cells to the proteasome inhibitor MG132 increases the efficiency of FIV-mediated gene delivery to TM cells in culture and in our MOCAS model, and results in more even distribution of transduction in the MOCAS TM. This strategy could become a useful adjunct for ocular gene therapy with lentiviral vectors.

## Supplementary Material

Supplement 1
