# Supplementary material for: Proteasome Inhibition Increases the Efficiency of Lentiviral Vector-Mediated Transduction of Trabecular Meshwork
Source: Invest Ophthalmol Vis Sci. 2018 May;59(1):298–310. doi: 10.1167/iovs.17-22074 (PMC5961099; doi:10.1167/iovs.17-22074)
Supplement: Supplement 1 [file iovs-58-14-33_s01-s02.pdf]

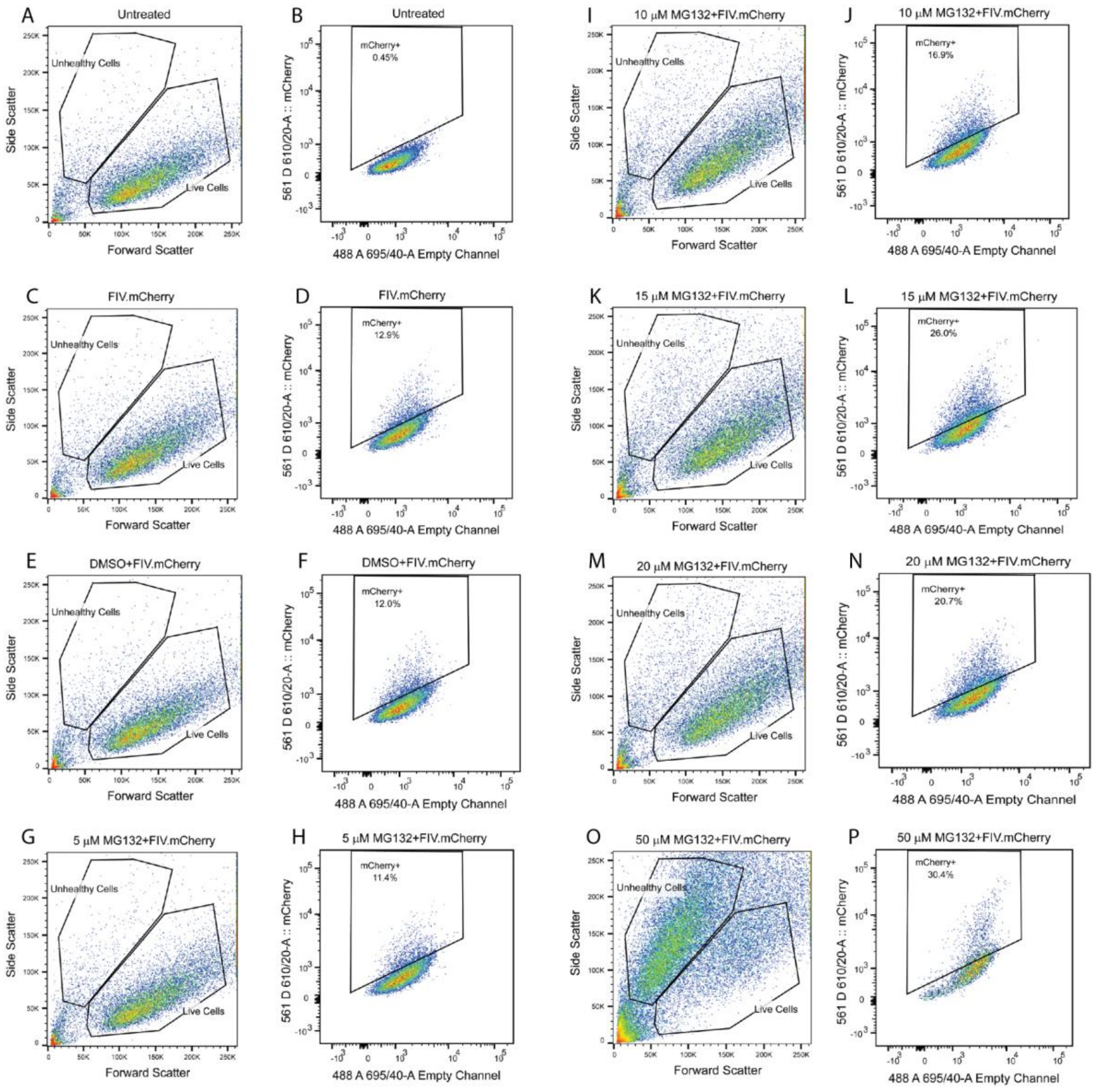

Figure S1. **Flow cytometry analysis of FIV.mCherry transduction enhancement** Panel A shows representative examples of the sorting for each of the groups, indicating the gating to separate normal and healthy cells. Panel B shows examples of the gating for mCherry+ cells. 3A, 3B: Control, untreated TM-1 cells; 3C, 3D: transduced with FIV.mCherry at a MOT of 25. Panels 3E to 3P are TM-1 cells transduced with FIV.mCherry at a MOT of 25 with different pretreatments. 3E, 3F: 0.5%DMSO; 3G, 3H: 5 μM MG132; 3I, 3J: 10 μM MG132; 3K, 3L: 15 μM MG132; 3M, 3N: 20 μM MG132; 3O, 3P: 50μM MG132.

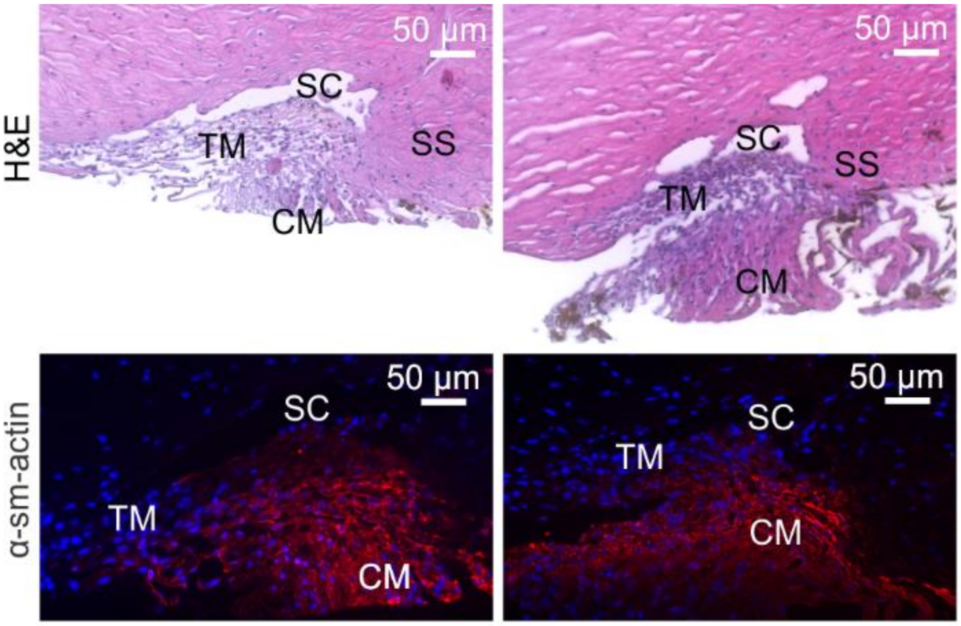

Figure S2. Histological analysis of TM outflow pathways from transduced MOCAS. H&E staining (upper panel) and α-sm-actin (Alexa 555 fluorophore) immunohistochemistry of consecutive sections (lower panel). Trabecular meshwork (TM) and Schlemm's canal (SC) are intact. At the posterior end of the TM close to its attachment to the scleral spur (SC), remnants of the anterior end of the longitudinal portion of the ciliary muscle (CM) that stain for α-sm-actin are present. CM remnants are rotated anteriorly/outwardly and are typically about 10 μm in length (left-hand panels). In some quadrants, longer muscle tips with a length of up to 100 μm were observed (right-hand panels). Paraffin sections of samples treated with 20 μM MG132 + 0.5% DMSO for 1 hr prior to transduction with high dose FIV (right-hand panels) or with FIV alone (left-hand panels).
